# Comprehensive care for childhood obesity in Brazilian municipalities

**DOI:** 10.11606/s1518-8787.2024058005632

**Published:** 2024-07-22

**Authors:** Maria Irene de Castro Barbosa, Laura Solléro de Paula, Elisabetta Recine

**Affiliations:** I Universidade de Brasília Faculdade de Ciências da Saúde Programa de Pós-Graduação em Nutrição Humana Brasília DF Brasil Universidade de Brasília. Faculdade de Ciências da Saúde. Programa de Pós-Graduação em Nutrição Humana. Brasília, DF, Brasil; II Universidade de Brasília Faculdade de Ciências da Saúde Programa de Iniciação Científica Brasília DF Brasil Universidade de Brasília. Faculdade de Ciências da Saúde. Programa de Iniciação Científica. Brasília, DF, Brasil; III Universidade de Brasília Faculdade de Ciências da Saúde Departamento de Nutrição Brasília DF Brasil Universidade de Brasília. Faculdade de Ciências da Saúde. Departamento de Nutrição. Brasília, DF, Brasil

**Keywords:** Comprehensive Health Care, Child Care, Childhood Obesity, Primary Health Care

## Abstract

**OBJECTIVE:**

To understand the potential and limits of care for childhood obesity from the perspective of comprehensiveness, in the context of Primary Health Care, in Brazilian municipalities.

**METHODS:**

A qualitative approach was adopted, with an electronic form of a dissertative nature being applied in 11 municipalities in the five Brazilian regions, derived from the four axes of comprehensiveness defined by Ayres (needs, purposes, articulations, and interactions).

**RESULTS:**

Among the strengths for comprehensive care, the following were observed: the provision of services at different levels of care; the relevance of intersectoral programs in the development of actions aimed at the multidimensionality of childhood obesity; the implementation of strategies for systematizing care and tools that encourage the expansion of dialogue and humanization; and intersectoral coordination to create appropriate responses to the expanded needs of children and their families. Limitations include: the centralization of actions in nutrition professionals and in the care sphere; the failure to prioritize childhood obesity in health agendas; and the lack of trained professionals to deal with the complexity of obesity.

**CONCLUSIONS:**

The findings suggest that child obesity care practices, in order to be transformative, need to be understood in the context of comprehensiveness. And this includes (re)thinking public policies, professional practices, and the organization of work processes so that they are, in fact, more inclusive, participatory, dialogical, humanized, supportive, fair, and, therefore, effective.

## INTRODUCTION

In Brazil, in 2022, data from the Ministry of Health’s Food and Nutrition Surveillance System indicated that the prevalence of overweight in children between 5 and 10 years of age, treated in primary care, was 25.37%^1^. This scenario has significant consequences, including: demand for care, greater risk of developing chronic and metabolic diseases, psychological problems, social and economic impacts, and living with excess weight throughout life^2-4^. Thus, care for childhood obesity requires processes that dialog with its multicausality^5^.

In Brazil, various government initiatives are aimed at tackling childhood obesity, including intersectoral strategies such as the Health at School Program (PSE), which created the Growing Up Healthy Program^6^. The “Instruction for the care of overweight and obese children and adolescents in Primary Health Care” was also made available to professionals^7^, and the implementation of the Strategy for the Prevention and Care of Childhood Obesity (PROTEJA) began^8^. In all these references and initiatives, the importance of practicing care from the perspective of comprehensiveness is emphasized.

Integrality in health is opposed to the fragmentation and intense specialization of health care, as it understands the user as a biopsychosocial being with rights to be respected^9,10^, and can be understood from four axes^9^. The axis of needs refers to welcoming people, making actions more flexible and looking at broader health needs; the axis of purposes emphasizes the integration and longitudinality of care; the axis of articulations focuses on collective work from an intersectoral and multi-professional perspective; and the axis of interactions refers to dialogical approaches and the articulation between practical and technical knowledge. These propositions indicate ways of expanding our understanding of the elements required to qualify care practices related to childhood obesity.

Thus, this study aims to answer: what are the limits and potentialities of childhood obesity care, aimed at children between 5 and 10 years old, from the perspective of comprehensive health care, in the context of Primary Health Care (PHC) in Brazilian municipalities?

## METHODS

A qualitative approach was adopted in order to grasp a set of meanings produced in human relationships, in health processes and practices that cannot be quantified^11^.

An electronic form was developed (Chart 1) with four axes that make up the concept of integrality in health^9^. The questionnaires were created using Google Forms and sent via e-mail.

**Chart 1:** Questionnaire to characterize the structuring of obesity care in children in different municipalities in Brazil, Brasília, 2020.

BLOCK 1 - NEEDS AXIS	
1	Is the reception of children with obesity in health services done via spontaneous and/or scheduled demand? Describe in general terms (activities, professionals involved) how reception takes place.
2	If there is a need and demand from the area, does the health team have the possibility of organizing new (or extra) activities related to preventing childhood obesity and/or caring for children with childhood obesity? Please comment on your answer regarding the possibility of new (or extra) activities.
3	Is there monitoring of the prevalence of childhood obesity in children between 5 and < 10 years old in the municipality? Please comment on your answer.
**BLOCK 2 – PURPOSES AXIS**	
1	In relation to the prevention of childhood obesity and the care of children with obesity, are promotion, prevention, treatment, and recovery actions integrated? Please comment on how this integration takes place (or why it is not integrated).
2	Do health services and teams consider the longitudinality of actions in the care of childhood obesity in the territory? How does longitudinality come about (or why doesn’t it happen)?
3	In actions to prevent childhood obesity and care for children with obesity, is there an explicit concern to protect children’s rights? Please give examples to illustrate your answer.
4	Is there an explicit approach to identifying, preventing, and acting on stigmatizing actions and practices in relation to childhood obesity and children with obesity? Please comment and/or give examples in relation to your answer.
5	Does the care of children with obesity also include extended needs, i.e. aspects other than weight loss and the adequacy of the type and quantity of food consumed? Please comment on how extended needs are met (or why they are not).
6	Are the prevention of childhood obesity and the care of children with obesity developed through co-responsible actions between different agents and sectors (e.g. health professionals, education professionals, managers, children and their guardians, etc.)? Please comment on how co-responsibility occurs (or why it doesn’t occur).
**BLOCK 3 – ARTICULATIONS AXIS**	
1	In the prevention of childhood obesity and the care of children with childhood obesity, is the planning of actions interdisciplinary, multiprofessional, intersectoral? Does each professional and/or area plan separately? Please give examples to illustrate your answer.
2	Do the different care agents (e.g. health professionals, education professionals, managers, children and their guardians, etc.) participate in defining actions to prevent childhood obesity and care for children with obesity? Please give examples to illustrate your answer.
**BLOCK 4 – INTERACTIONS AXIS**	
1	Do practices for preventing childhood obesity and caring for children with obesity provide for: the exercise of an expanded clinic; the establishment of dialogic relationships and the exercise of qualified listening? Please comment, give examples of situations and/or actions that illustrate your answer.
2	In actions to prevent childhood obesity and care for children with obesity, are there strategies that allow the technical knowledge of professionals (health, education, etc.) and the knowledge, practices, and values of children with obesity and their guardians to interact? Please comment and/or give examples to illustrate your answer.

Source: The authors.

The audience for this research participated in the monitoring of the Growing Up Healthy Program - a set of actions carried out under the PSE to tackle childhood obesity^6^ - in 2018, promoted by the General Coordination of Food and Nutrition of the Ministry of Health (CGAN/MS).

The inclusion criteria were: representation of at least one municipality per region of Brazil and the choice of municipalities with the highest percentage of affirmative responses, in the CGAN/MS monitoring, to themes that referred to the axes of integrality in health. In the end, 11 of the 180 municipalities monitored took part, covering the five Brazilian regions (Chart 2). An exception was the municipality of Aracaju, which, despite not having answered the CGAN/MS monitoring questionnaire, was included because it was implementing the Line of Care for Overweight and Obesity in Children and Adolescents^12^, which is a differentiated strategy for implementing comprehensive care.

**Chart 2:** Municipalities participating in the survey, Brasília, 2022.

Municipality (State)	Region of Brazil	Percentage of affirmative responses to the CGAN questionnaire on elements of comprehensive care for childhood obesity
Paraúna (Goiás)	Midwest	100% (n = 11)
Serrolândia (Bahia)	North East	90.9% (n = 10)
Aracaju (Sergipe)		Non-respondent*
Caaporã (Paraíba)		100% (n = 11)
Barcarena (Pará)	North	100% (n = 11)
Jerônimo Monteiro (Espírito Santo)	South East	81.8% (n = 9)
Barretos (São Paulo)		100% (n = 11)
Arroio do Tigre (Rio Grande do Sul)	South	90.9% (n = 10)
Foz do Iguaçu (Paraná)		72.7% (n = 8)
Chapecó (Santa Catarina)		100% (n = 11)
Curitiba (Paraná)		72.7% (n = 8)

*Municipality included due to the implementation process of the Line of Care for Overweight and Obesity in Children and Adolescents^11^

CGAN: General Coordination of Food and Nutrition.

Source: Coordenação-Geral de Alimentação e Nutrição (CGAN/MS)

The municipalities were invited via the information recorded on the CGAN/MS form and publications on official websites. The respondents were PHC nutritionists, coordinators of the Expanded Family Health Center, and reference professionals in childhood obesity care.

The data was obtained between May and July 2021 and analyzed using the Thematic Analysis (TA) method^13^, which makes it possible to identify, analyze, and report patterns in discourses.

Participation was preceded by acceptance of the Electronic Free and Informed Consent Form. This study was approved by the Research Ethics Committee of the University of Brasília (CEP-FS/UnB) and by the Research Ethics Committee of the Federal District Health Department (CEP-Fepecs) (CAE 33905320.5.3001.5553).

## RESULTS AND DISCUSSION

The results of the study have been organized into themes and their derivations (codes), as shown in the figure and analyses below.

**Figure:**
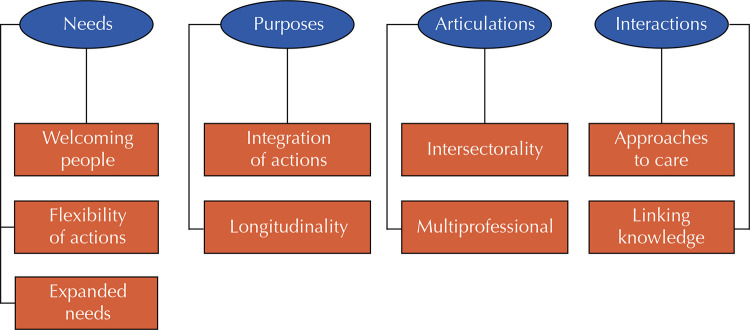
Themes (rounded figure) and their respective codes (rectangles) produced when applying the TA method13.

### Needs Axis

In the 11 participating Brazilian municipalities, children with obesity were seen by spontaneous and scheduled demand in six municipalities (four in the South, one in the Northeast, and one in the Center-West); only by scheduled demand in three (two in the Northeast and one in the Southeast) and by spontaneous demand in two (in the North and Southeast).

The southern region presented more details on how reception is carried out and pointed out projects and flows created to deal with childhood obesity, reinforcing that prioritizing the agenda in the health service expands and strengthens the different forms of reception and their consequences.

In the different regions, the reception via programmed demand took place through: periodic anthropometric evaluations carried out in the school environment, within the scope of the PSE, and/or in health units linked to income transfer programs; and the active search by community health agents for children with obesity. Thus, initial access to care occurred not only through the population’s identification of the need for “care”, but also through actions in the school environment.

Dornelles et al.^14^ listed, among the perceptions of health professionals about the role of society and the family in caring for overweight and obese children in the Unified Health System (SUS), that it is difficult for caregivers to recognize it as a health demand to be cared for. Thus, if reception is restricted exclusively or primarily to spontaneous demand, there may be a limitation in identifying the needs of the territory. Furthermore, there is a need for the issue of childhood obesity to be increasingly prioritized, with actions that problematize and reflect this scenario and encompass the different players involved in care^14^.

The Health at School (PSE) and Growing Up Healthy programs, developed under the coordination of PHC, were essential for “provoking” changes in the work process of health teams, through the diagnosis and monitoring of childhood obesity cases, and the development of collective activities, such as health education groups. Studies^15,16^ reinforce that intersectoral coordination between health and education - structured by dialogical governance mechanisms, with integration of efforts that minimize the overlapping of actions - is fundamental for effective prevention and control of childhood obesity.

Methods for monitoring the prevalence of childhood obesity have become essential for taking in children with alterations in their nutritional status, despite limitations in terms of: the restriction of human resources and the high demand for work; the insufficient entry of data on nutritional status in the SUS information systems; and the discontinuity and low coverage of assessments of children’s nutritional status.

We observed that the most detailed reports of monitoring were found in municipalities in the southern region that managed to implement protocols/flows, and even the Line of Care for Overweight and Obesity (LCSO), linked to childhood obesity care. A study by Ribeiro et al.^17^, carried out in Sergipe, points out that the power of the care line requires attributes such as: proactivity and continuity of care; co-responsibility of the actors involved in the process; understanding obesity as a dysfunction that requires treatment; and offering promotion and treatment actions from an intersectoral perspective. Faced with comprehensive care, these elements can culminate in the creation of a sensitive space to respond to the demands of the territory^9^.

By understanding how activities are designed to meet the health needs of municipalities, it was observed that the versatility of actions in the field of childhood obesity is connected to both the perception of health professionals and the demands of users. However, Silva et al.^18^ point out that the organization of care requires a connection between different agents and also co-responsibility between them and the services. One strategy cited which has strengthened the approach to childhood obesity in PHC is matrix support for the Family Health Strategy (ESF) teams.

In all the municipalities, flexibility was identified in organizing activities related to the prevention and/or care of children with childhood obesity, although in some cases these occurred irregularly and were centered on the nutritionist. Among these were health education groups linked or not to the Health Academies, workshops, lectures, the production of information materials, and interviews in different media.

It was also analyzed whether there was an expanded understanding of the demands of the subjects, based on the ability to adequately contextualize these actions to the different realities^19^. This refers to the ability of municipalities to produce actions that consider the historicity of children and their families, which goes beyond the physiological condition of obesity.

In all the municipalities, it was reported that the guidelines are not restricted to weight loss and dietary adequacy. The need to consider mental health, active listening to children and family members and attention to social vulnerabilities was also considered. Different publications^20^ emphasize that impactful strategies for managing childhood obesity involve the social and physical context and the child’s eating environment. However, although all the municipalities confirmed the importance of considering different factors in the care of childhood obesity, some justifications reinforced behaviors centered on food consumption.

It is important to emphasize that comprehensive care is linked to strengthening a more just and supportive society^19^. In the context of childhood obesity, stigmatizing actions are part of children’s daily lives. Poulain^23^ defines them as the social devaluation and defamation of children due to their excess body weight, which can lead to negative attitudes, stereotypes and discrimination. Faced with the need for sensitive spaces to respond to the demands of the territories studied, we questioned the implementation of activities to identify and prevent stigmatizing practices. Four municipalities - one in the Central-West region, one in the Northeast, one in the Southeast and one in the South - reported carrying out such activities. The activities were similar and were developed in partnership between the Departments of Education and Health, in the school environment, or in professional training. According to one of the reports, the school seems to be a strategic locus, due to its ability to strengthen bonds, as children are there on a daily basis.

However, only one municipality in the southern region reported that stigmas were addressed individually, albeit less frequently, when compared to dialogues on the same subject held in group activities. Two municipalities said they did not address this issue; two said there was nothing explicit/official and it depended on professional practice; and three said they had no such information.

In the context of guaranteeing rights as a prerogative of comprehensive healthcare^10^, despite the small number of descriptions of explicit concern with protecting children’s rights, responses from the Midwest, South, and Southeast regions made contributions. In the first, the right materialized in access to “information in an easy way for both children and caregivers, through various means of communication (radio, social networks, other communication applications), contributing to awareness and promotion of health and well-being; promotion of leisure and physical activities” (municipality in the Midwest region). In the municipality in the South, one of the facilities that helps to guarantee this is the Social Assistance Reference Center (CRAS), which acts on the social vulnerabilities of families, as well as raising the awareness of health professionals about the increasing prevalence of childhood obesity and the need to intervene. In the Southeast, one of the reports pointed to the difficulty of accessing health facilities and continuity of care as a limitation.

### Purposes Axis

Only one municipality in the Southeast and one in the South described the integration of health promotion and protection, treatment, and recovery activities. The municipality in the Southeast has an obesity outpatient service, which establishes communication with the primary health care units, by referring the child and making a counter-referral to PHC. All care related to childhood obesity is channeled to an obesity outpatient clinic.

The municipality in the southern region has a different perspective on the organization and integration of care, since it has implemented the LCSO, in which various health/social facilities are linked. The care provided in the other municipalities is of an assistance nature, with priority given to clinical care, developed mainly by nutritionists. Actions to promote children’s health and prevent childhood obesity take place separately and are concentrated in the Growing Up Healthy Program. Such disarticulation of actions, making them “loose and unaligned” (municipality in the Southeast region), “discontinuous and of punctual and isolated execution” (municipality in the South region), was justified by the lack of professionals.

The fragmentation of care, with health care performed through a sum of specialized and disassociated actions, was listed by Moura and Recine^24^ as one of the obstacles to the practice of integrality in the SUS. In order for health actions to operate in an integrated manner, coordinated by a flow of care, they must be based on longitudinal care, achieved by the continuity, permanence and consistency of care, accompanying the transformations of health practices^21^.

According to Rajjo et al.^20^, significant results in childhood obesity care are the result of integrated and longitudinal programs to manage children’s lifestyles. Three participating municipalities (Midwest, Northeast, and South regions) in this study said that the practical exercise of longitudinal care is directly dependent on the bond created and the interest of the child and family in continuing the care.

Other factors that made it difficult to achieve longitudinality were the high demand and small number of professionals, and the ESF’s failure to understand the logic of primary care^25^, as the coordinator of care.

### Articulations Axis

This study adopts intersectorality and multi-professional coordination as components of collective work, which are essential for the exercise of comprehensive care, as well as interdisciplinarity - although this was not evidenced in the municipalities’ responses.

Interdisciplinarity seeks to find the links that connect one area and its historicized foundations to another, seeking to broaden understandings and practices^26^. Multiprofessional means the presence of different professionals working on shared actions^25^. Thus, multi-professional coordination may or may not lead to transformations in professional practices, unlike the interdisciplinary approach. Finally, intersectorality is defined as actions in which different social sectors collaborate to achieve a common goal, through close coordination of their contributions.^27^

The participants’ understanding of interdisciplinarity was confused with its multiprofessional nature. In municipalities in the Midwest and Northeast regions, although they mentioned that there is interdisciplinary and multiprofessional planning, their justifications only covered the multiprofessional perspective.

One of the municipalities in the Northeast region pointed out that planning “actions with intersectoral and multi-professional support are more likely to achieve the expected results, as each professional involved will add to the fight against obesity”. The collective work involved team discussions of cases, therapeutic groups and matrix support meetings.

On the other hand, the Northern region made it clear that multi-professional work is not always enough, as planning can be disjointed. The lack of qualified and regularly trained multi-professional teams is one of the most frequent barriers to comprehensive care for overweight children^20,28^.

The participation of education and health professionals, children, and their guardians in defining actions aimed at childhood obesity was another topic studied. It was observed that coordination and planning are carried out by health professionals. In one municipality in the Southeast, only the nutritionist takes part in planning. The presence of education professionals was mentioned in the Midwest, Northeast, and South regions during planning, execution and approval by the Education Department managers.

Intersectoral coordination took place mainly with the education sector, through anthropometric assessments and educational activities. As an exception, one of the municipalities in the southern region praised the importance of the Municipal Council for Food and Nutritional Security as a facilitator of dialogue between sectors - health, education, social assistance, sport, and food and nutritional security - and its constitution, which includes civil society.

In nine municipalities, it was difficult to articulate and prioritize childhood obesity on the health agenda. Non-prioritization was due to: lack of implementation of protocols and flows in the care network; insufficient human resources, prioritization of individualized care for more critical cases, concentration of prevention actions in the PSE; and lack of interest on the part of health professionals. This scenario expresses insufficient engagement by the parties involved in childhood obesity care. Thus, it emphasizes that integrality faces barriers in terms of governance, given the incipient communication and cooperation between the different sectors and spheres of management^22^. Where care for children with obesity has been prioritized, it has been strengthened with the implementation of the LCSO.

The engagement of the parties involved is also linked to co-responsibility in care. Although the planning of actions is restricted to a few groups, co-responsibility occurs through the work of managers and health and education professionals. Municipalities in the Midwest, Northeast, North, one in the Southeast, and one in the South mentioned the active participation of children and family members in the care process.

This perspective corroborates comprehensive practices, since there is recognition of the need to create a bond, making the individuals who take part in this long process accountable, considering the ways in which children and their families express their health needs^10^.

### Interactions Axis

Different approaches used by health professionals, in the individual and collective spheres, can exercise aspects of comprehensive care that include the need to: listen more, to understand singularities, subjectivities, and the context in which individuals and collectivities are immersed; see more, be attentive to gestures, to the gaze; and touch more, not just where it hurts, but where there is discomfort, concern, as a power for care^9^.

In all the municipalities studied, the exercise of qualified listening and the expanded clinic took place at both an individual and collective level. One municipality in the Northeast and one in the South reported that these strategies “help to uncover children’s needs in a broader way” (municipality in the Northeast), “to define their treatment in the best way” (municipality in the South).

These strategies can strengthen comprehensive practices, since they encourage active participation. Excerpts 1 and 2 describe ways in which the approaches have been applied in health services, both in care practice and in professional training:

Excerpt 1

Qualified listening and dialogic relationships are present in both group and individual consultations, always paying attention to the individuality of each patient and their demands. The extended clinic provides medical care, as well as occasional nutrition and psychology consultations, which serve to understand the reality of each family and provide guidance according to the demands identified and raised by the children and families (municipality in the southern region).

Excerpt 2

(...) the aforementioned concepts [qualified listening and expanded clinic] (...) are widely discussed at professional meetings, especially with the nutritionists who are the care coordinators at the LCSO. These professionals are responsible for training the teams in how to care for users with obesity (municipality in the Northeast region).

The active role of different subjects in child obesity care actions is essential, through the establishment of dialogic relationships. According to Freire^29^, these interactions are moments to reflect on the problems experienced, in order to overcome them, considering the different knowledge and perspectives of those who participate in health care.

All the participants said that there is an exchange of technical and practical knowledge but with different characteristics. A municipality in the North region mentioned that this practice happens occasionally and depends on the professional’s initiative. One municipality in the Southeast stressed the need for management support to raise awareness of the importance of these exchanges.

Only the southern region provided details of these dialogues. Most of them are the result of group activities led by different health professionals, establishing intersectoral partnerships with or without the participation of children and their guardians. The exchanges in these spaces take place in a dialogical way and are powerful in understanding the factors that impact on the development of obesity in a specific group, contributing to the expansion of resolution. The protagonism of children stood out, despite the fact that they are usually excluded and silenced. According to Ehrich et al.^30^, including children actively can improve their knowledge, self-confidence, imagination and trust in health professionals, contributing to their autonomy and the effectiveness of comprehensive care.

Regarding the study’s limitations, it should be noted that incomplete questionnaires were received and there were difficulties in contacting the Central-West and North regions. We understand that the pandemic period was a factor that limited the detailing of actions and the implementation of activities developed in the context of childhood obesity.

## CONCLUSIONS

Comprehensiveness is a horizon to be reached in the practice of childhood obesity care. It is desired as a guideline for the organization of services in the municipalities studied but it still faces challenges in its implementation.

As strengths for comprehensive care, the following were observed: the provision of services at different levels of care; the relevance of the PSE and the Growing Up Healthy Program in developing actions aimed at the multidimensionality of childhood obesity; strategies for systematizing care, such as the implementation of the LCSO and the expansion of dialogue and humanization in health practices; and intersectoral coordination to create appropriate responses to the expanded needs of children and their families.

Finally, in order for childhood obesity care to be able to bring about changes in the reality of different subjects, it needs to be understood in its complexity, and this is directly related to looking at integrality in health and its dynamism. This includes (re)thinking public policies, professional practices, and work process organizations so that they are, in fact, more inclusive, participatory, dialogical, humanized, supportive, fair, and, therefore, effective. In this sense, there is a need to mobilize different sectors/spaces/subjects in favor of this agenda.
