# Supplementary material for: Comprehensive care for childhood obesity in Brazilian municipalities
Source: Rev Saude Publica. 2024 Jul 22;58:33. doi: 10.11606/s1518-8787.2024058005632 (PMC11319039; doi:10.11606/s1518-8787.2024058005632)
Supplement: Translation

# Translation: A integralidade no cuidado da obesidade infantil em municípios brasileiros

**Authors:** Maria Irene de Castro Barbosa, Laura Solléro de Paula, Elisabetta Recine

**Affiliations:** I Brasil Universidade de Brasília. Faculdade de Ciências da Saúde. Programa de Pós-Graduação em Nutrição Humana. Brasília, DF, Brasil; II Brasil Universidade de Brasília. Faculdade de Ciências da Saúde. Programa de Iniciação Científica. Brasília, DF, Brasil; III Brasil Universidade de Brasília. Faculdade de Ciências da Saúde. Departamento de Nutrição. Brasília, DF, Brasil

**Keywords:** Integralidade em Saúde, Cuidado da Criança, Obesidade Infantil, Atenção Primária à Saúde

## Abstract

**OBJETIVO:**

Compreender potencialidades e limites do cuidado em obesidade infantil na perspectiva da integralidade, no contexto da Atenção Primária à Saúde, em municípios brasileiros.

**MÉTODOS:**

Adotou-se a abordagem qualitativa, com aplicação, em 11 municípios das cinco regiões brasileiras, de um formulário eletrônico de caráter dissertativo, derivado dos quatro eixos da integralidade definidos por Ayres (necessidades, finalidades, articulações e interações).

**RESULTADOS:**

Dentre as potências para o cuidado integral, observou-se: a oferta de serviços em diferentes níveis de atenção; a relevância de programas intersetoriais no desenvolvimento de ações voltadas à multidimensionalidade da obesidade infantil; a implementação de estratégias de sistematização do cuidado e ferramentas que estimulem a ampliação do diálogo e a humanização; e a articulação intersetorial para criação de respostas adequadas às necessidades ampliadas das crianças e suas famílias. Enquanto limitações, estão: a centralização das ações nos profissionais nutricionistas e no âmbito assistencial; a não priorização da obesidade infantil nas agendas da saúde; e a insuficiência dos profissionais capacitados para lidar com a complexidade da obesidade.

**CONCLUSÕES:**

Os achados sugerem que as práticas no cuidado da obesidade infantil, para que sejam transformadoras, precisam ser entendidas no âmbito da integralidade. E isso inclui (re)pensar políticas públicas, práticas profissionais, organização dos processos de trabalho para serem, de fato, mais inclusivas, participativas, dialógicas, humanizadas, solidárias, justas e, portanto, eficazes.

## INTRODUÇÃO

No Brasil, em 2022, dados do Sistema de Vigilância Alimentar e Nutricional do Ministério da Saúde indicaram que a prevalência de excesso de peso em crianças entre 5 e 10 anos, atendidas na atenção primária, era de 25,37%^1^. Esse panorama traz consequências significativas, incluindo: demanda por ações de cuidado, maior risco de desenvolvimento de doenças crônicas e metabólicas, acometimentos psicológicos, impactos sociais e econômicos e convivência com o excesso de peso ao longo da vida^2-4^. Assim, a atenção à obesidade infantil requer processos que dialoguem com sua multicausalidade^5^.

Diferentes iniciativas governamentais buscam o enfrentamento da obesidade infantil, inclusive estratégias intersetoriais como o Programa Saúde na Escola (PSE), que criou o Programa Crescer Saudável^6^. Também foi disponibilizado aos profissionais o “Instrutivo para o cuidado da criança e do adolescente com sobrepeso e obesidade no âmbito da Atenção Primária à Saúde”^7^, e iniciada a implementação da Estratégia de Prevenção e Atenção à Obesidade Infantil (Proteja)^8^. Em todas essas referências e iniciativas, está ressaltada a importância da prática do cuidado na perspectiva da integralidade.

A integralidade em saúde contrapõe-se à fragmentação e à intensa especialização da atenção à saúde, pois compreende o usuário enquanto um ser biopsicossocial com direitos a serem respeitados^9,10^, e pode ser compreendida a partir de quatro eixos^9^. O eixo das necessidades refere-se ao acolhimento, à flexibilização de ações e ao olhar sobre as necessidades ampliadas em saúde; o das finalidades enfatiza a integração e a longitudinalidade do cuidado; o das articulações atenta-se para o trabalho coletivo na perspectiva intersetorial e multiprofissional; e o das interações remete às abordagens dialógicas e à articulação entre saberes práticos e técnicos. Tais proposições sinalizam caminhos para expandir a compreensão sobre elementos requeridos na qualificação das práticas de cuidado relacionadas à obesidade infantil.

Assim, este estudo objetiva responder: quais os limites e as potencialidades do cuidado em obesidade infantil, destinado a crianças entre 5 e 10 anos, na perspectiva da integralidade em saúde, no contexto da Atenção Primária à Saúde (APS) em municípios brasileiros?

## MÉTODOS

Adotou-se a abordagem qualitativa visando apreender um conjunto de significados produzidos nas relações humanas, em processos e práticas de saúde que apresentam caráter não quantificável^11^. Desenvolveu-se um formulário eletrônico (Quadro 1) com quatro eixos que compõem o conceito de integralidade em saúde^9^. Os questionários foram elaborados no *Google Forms* e encaminhados via endereço eletrônico.

**Quadro 1:** Roteiro de perguntas para caracterização da estruturação do cuidado em obesidade em crianças em diferentes municípios do Brasil, Brasília, 2020.

BLOCO 1 - EIXO DAS NECESSIDADES	
1	O acolhimento nos serviços de saúde de crianças com obesidade é feito via demanda espontânea e/ou programada? Descreva em linhas gerais (atividades, profissionais envolvidos) como o acolhimento ocorre.
2	Caso haja necessidade e demanda do território, a equipe de saúde tem possibilidade de organizar atividades novas (ou extras) relacionadas à prevenção da obesidade infantil e/ou ao cuidado de crianças com obesidade infantil? Por favor, comente sua resposta a respeito da possibilidade de atividades novas (ou extras).
3	Há monitoramento da prevalência de obesidade infantil em crianças entre 5 e < 10 anos no município? Por favor, comente a sua resposta.
**BLOCO 2 - EIXO DAS FINALIDADES**	
1	Em relação à prevenção da obesidade infantil e ao cuidado de crianças com obesidade, as ações de promoção, prevenção, tratamento e recuperação são integradas? Por favor, comente como se dá essa integração (ou por que não são integradas).
2	Os serviços e as equipes de saúde consideram a longitudinalidade das ações no cuidado da obesidade infantil no território? Como a longitudinalidade se concretiza (ou por que não acontece)?
3	Nas ações de prevenção da obesidade infantil e do cuidado das crianças com obesidade, há uma preocupação explícita de proteger os direitos das crianças? Por favor, dê exemplos que ilustrem sua resposta.
4	Há uma abordagem explícita para identificar, prevenir e atuar sobre ações e práticas estigmatizantes em relação à obesidade infantil e à criança com obesidade? Por favor, comente e/ou dê exemplos em relação à sua resposta.
5	O cuidado com as crianças com obesidade também inclui as necessidades ampliadas, ou seja, outros aspectos para além de perda de peso, adequação de tipo e quantidade de alimentos consumidos? Por favor, comente como se dá o atendimento às necessidades ampliadas (ou por que não ocorre).
6	A prevenção da obesidade infantil e o cuidado da criança com obesidade são desenvolvidos por meio de ações corresponsáveis, entre diferentes agentes e setores (por exemplo, profissionais da saúde, profissionais da educação, gestores, crianças e seus responsáveis etc.)? Por favor, comente como se dá a corresponsabilidade (ou por que ela não ocorre).
**BLOCO 3 - EIXO DAS ARTICULAÇÕES**	
1	Na prevenção da obesidade infantil e no cuidado das crianças com obesidade infantil, o planejamento de ações é interdisciplinar, multiprofissional, intersetorial? Cada profissional e/ou área faz seu planejamento separadamente? Por favor, dê exemplos que ilustrem a sua resposta.
2	Os diferentes agentes do cuidado (por exemplo, profissionais da saúde, profissionais da educação, gestores, crianças e seus responsáveis etc.) participam da definição das ações de prevenção da obesidade infantil e do cuidado das crianças com obesidade? Por favor, dê exemplos que ilustrem a sua resposta.
**BLOCO 4 - EIXO DAS INTERAÇÕES**	
1	As práticas de prevenção da obesidade infantil e de cuidado de crianças com obesidade preveem: o exercício da clínica ampliada; o estabelecimento de relações dialógicas e o exercício da escuta qualificada? Por favor, comente, exemplifique situações e/ou ações que ilustrem a sua resposta.
2	Nas ações de prevenção da obesidade infantil e do cuidado das crianças com obesidade, há estratégias que permitem que os conhecimentos técnicos dos profissionais (da saúde, da educação etc.) e os conhecimentos, práticas e valores das crianças com obesidade e seus responsáveis dialoguem? Por favor, comente e/ou dê exemplos que ilustrem sua resposta.

Fonte: As autoras.

O público desta pesquisa participou do monitoramento do Programa Crescer Saudável – conjunto de ações realizadas no âmbito do PSE para o enfrentamento da obesidade infantil^6^ –, em 2018, promovido pela Coordenação-Geral de Alimentação e Nutrição do Ministério da Saúde (CGAN/MS).

Os critérios de inclusão foram: representação de, pelo menos, um município por região do Brasil e opção pelos municípios que apresentaram maior percentual de respostas afirmativas, no monitoramento da CGAN/MS, para temas que remetiam aos eixos da integralidade em saúde. Ao final, dos 180 municípios monitorados, participaram 11, contemplando as cinco regiões brasileiras (Quadro 2). Como exceção, está a adição do município de Aracaju, que, apesar de não ter respondido ao questionário de monitoramento da CGAN/MS, foi incluído por estar implementando a Linha de Cuidado para o Sobrepeso e Obesidade de Crianças e Adolescentes^12^, sendo estratégia diferenciada para efetivação do cuidado integral.

**Quadro 2:** Municípios participantes da pesquisa, Brasília, 2022.

Município (Estado)	Região do Brasil	Percentual de respostas afirmativas sobre elementos da integralidade no cuidado da obesidade infantil contidas no questionário da CGAN
Paraúna (Goiás)	Centro-Oeste	100% (n = 11)
Serrolândia (Bahia)	Nordeste	90,9% (n = 10)
Aracaju (Sergipe)		Não respondente*
Caaporã (Paraíba)		100% (n = 11)
Barcarena (Pará)	Norte	100% (n = 11)
Jerônimo Monteiro (Espírito Santo)	Sudeste	81,8% (n = 9)
Barretos (São Paulo)		100% (n = 11)
Arroio do Tigre (Rio Grande do Sul)	Sul	90,9% (n = 10)
Foz do Iguaçu (Paraná)		72,7% (n = 8)
Chapecó (Santa Catarina)		100% (n = 11)
Curitiba (Paraná)		72,7% (n = 8)

*Município incluído devido ao processo de implementação da Linha de Cuidado para o Sobrepeso e Obesidade de Crianças e Adolescentes^11^

CGAN: Coordenação-Geral de Alimentação e Nutrição.

Fonte: Coordenação-Geral de Alimentação e Nutrição (CGAN/MS)

O convite aos municípios foi feito por meio das informações registradas no formulário da CGAN/MS e de publicações em *sites* oficiais. Os respondentes foram nutricionistas da APS, coordenadores(as) do Núcleo Ampliado de Saúde da Família e profissionais de referência no cuidado da obesidade infantil.

Os dados foram obtidos entre maio e julho de 2021 e analisados pelo método da Análise Temática (AT)^13^, que permite identificar, analisar e relatar padrões em discursos.

A participação precedeu o aceite ao Termo de Consentimento Livre e Esclarecido Eletrônico. Esta pesquisa foi aprovada pelo Comitê de Ética em Pesquisa da Universidade de Brasília (CEP-FS/UnB) e pelo Comitê de Ética em Pesquisa da Secretaria do Estado de Saúde do Distrito Federal (CEP-Fepecs) (CAE 33905320.5.3001.5553).

## RESULTADOS E DISCUSSÃO

Os resultados do estudo foram organizados em temas e suas derivações (códigos), conforme Figura e análises, estão apresentadas a seguir.

**Figura:** Temas (figura de formato arredondado) e seus respectivos *códigos* (retângulos) produzidos na aplicação do método da AT13.

### Eixo das Necessidades

Nos 11 municípios brasileiros participantes, o acolhimento a crianças com obesidade na APS ocorreu via demanda espontânea e programada em seis municípios (quatro da região Sul, um da região Nordeste e o da região Centro-Oeste); somente por demanda programada em três (dois da região Nordeste e um da Sudeste) e por demanda espontânea em dois deles (regiões Norte e Sudeste).

A região Sul apresentou maior detalhamento sobre como a acolhida é realizada e sinalizou projetos e fluxos criados para atendimento da obesidade infantil, reforçando que a priorização da agenda no serviço de saúde amplia e fortalece as diferentes formas de acolhimento e seus desdobramentos.

Nas diferentes regiões, o acolhimento via demanda programada decorreu por meio: das avaliações antropométricas periódicas desenvolvidas no ambiente escolar, no âmbito do PSE, e/ou nas unidades de saúde vinculadas a programas de transferência de renda; e da busca ativa dos agentes comunitários de saúde por crianças com obesidade. Assim, o acesso inicial ao cuidado ocorreu não somente a partir da identificação pela população ligada à necessidade de “cuidar”, mas também via ações no ambiente escolar.

Dornelles et al.^14^ elencaram, dentre as percepções dos profissionais de saúde acerca do papel da sociedade e da família na atenção ao sobrepeso e obesidade infantil no Sistema Único de Saúde (SUS), que há uma dificuldade dos cuidadores de a reconhecerem como uma demanda de saúde a ser cuidada. Assim, caso o acolhimento se restrinja exclusiva ou prioritariamente via demanda espontânea, é possível que haja limitação de identificação das necessidades do território. Ademais, percebe-se a necessidade de que a pauta da obesidade infantil seja cada vez mais priorizada, com ações que problematizem e reflitam este cenário e englobem os diferentes atores que participam do cuidado^14^.

Os programas Saúde na Escola e Crescer Saudável, desenvolvidos sob a coordenação da APS, foram essenciais para “provocar” desdobramentos no processo de trabalho das equipes de saúde, por meio do diagnóstico e monitoramento de casos de obesidade infantil, e desenvolvimento de atividades coletivas, como grupos de educação em saúde. Estudos^15,16^ reforçam que a articulação intersetorial entre a saúde e educação – estruturada por mecanismos de governança dialógicos, com integração de esforços que minimizem a sobreposição de ações – é fundamental para a efetividade da prevenção e do controle da obesidade infantil.

Os métodos de monitoramento da prevalência da obesidade infantil tornaram-se fundamentais para acolher crianças com alterações no estado nutricional, apesar das limitações quanto: à restrição de recursos humanos e alta demanda de trabalho; à insuficiência no lançamento de dados sobre o estado nutricional nos sistemas de informação do SUS; e à descontinuidade e baixa cobertura das avaliações do estado nutricional das crianças.

Observamos que os relatos mais detalhados de monitoramento estiveram presentes em municípios da região Sul que conseguiram implementar protocolos/fluxos, e até mesmo a Linha de Cuidado do Sobrepeso e Obesidade (LCSO), atrelados ao cuidado da obesidade infantil. Estudo de Ribeiro et al.^17^, desenvolvido em Sergipe, ressalta que a potência da linha de cuidado requer atributos como: proatividade e continuidade no cuidado; corresponsabilização de atores envolvidos no processo; compreensão da obesidade como disfunção que demanda tratamento; e oferta de ações de promoção e tratamento na perspectiva intersetorial. Diante do cuidado integral, tais elementos podem culminar na criação de espaço sensível para responder às demandas do território^9^.

Ao compreender como as atividades em torno das necessidades de saúde dos municípios são pensadas, observou-se que a versatilidade das ações no âmbito da obesidade infantil está conectada tanto à percepção dos profissionais de saúde quanto às demandas dos usuários. Contudo, Silva et al.^18^ ressaltam que a organização do cuidado requer conexão entre diferentes agentes e, ainda, a corresponsabilização entre estes e os serviços. Uma estratégia citada, que fortaleceu o olhar para a obesidade infantil na APS, foi o matriciamento junto às equipes de Estratégia Saúde da Família (ESF).

Em todos os municípios, foi identificada flexibilidade para organizar atividades relacionadas à prevenção e/ou ao cuidado de crianças com obesidade infantil, ainda que, em alguns casos, estas ocorram de maneira irregular e centralizadas na figura do nutricionista. Dentre estas, foram elencados os grupos de educação em saúde vinculados ou não às Academias da Saúde, oficinas, palestras, produção de materiais informativos, e realização de entrevistas em diferentes meios de comunicação.

Analisou-se ainda se há apreensão ampliada das demandas dos sujeitos, a partir da habilidade de contextualizar adequadamente essas ações às diferentes realidades^19^. Isto remete à capacidade dos municípios de produzir ações que considerem a historicidade das crianças e suas famílias, que ultrapassa a condição fisiológica da obesidade.

Em todos os municípios, foi relatado que as orientações não se restringem à perda de peso e à adequação alimentar. Ponderou-se, inclusive, a necessidade de considerar a saúde mental, escuta ativa das crianças e familiares e atenção às vulnerabilidades sociais. Diferentes publicações^20^ salientam que estratégias de impacto para o manejo da obesidade infantil envolvem o contexto social e físico e o ambiente alimentar da criança. Entretanto, ainda que todos os municípios tenham confirmado a importância de considerar diferentes fatores no cuidado da obesidade infantil, algumas justificativas reforçaram condutas centralizadas no consumo alimentar.

É importante ressaltar que o cuidado integral se vincula ao fortalecimento de uma sociedade mais justa e solidária^19^. No âmbito da obesidade infantil, as ações estigmatizantes fazem parte do cotidiano das crianças. Poulain^23^ as define como a desvalorização social e difamação das crianças devido ao excesso de peso corporal, podendo levar a atitudes negativas, estereótipos e discriminação. Diante da necessidade de espaços sensíveis para responder às demandas dos territórios estudados, questionamos a realização de atividades de identificação e prevenção de práticas estigmatizantes. Quatro municípios – um da região Centro-Oeste, um da Nordeste, um da Sudeste e um da Sul – relataram desenvolvê-las. As atividades foram semelhantes e desenvolvidas em parceria entre as Secretarias de Educação e Saúde, no âmbito escolar, ou em capacitações profissionais. Segundo um dos relatos, a escola parece ser um lócus estratégico, pela capacidade de fortalecer vínculos, pois as crianças estão ali cotidianamente.

Contudo, apenas um município da região Sul relatou haver abordagem sobre estigmas no âmbito individual, mesmo que com menor frequência, quando comparada a diálogos com a mesma temática realizados em atividades grupais. Dois municípios citaram não abordar essa temática; dois disseram que não há nada explícito/oficial e depende da prática profissional; e três relataram não ter tal informação.

No contexto da garantia de direitos enquanto uma prerrogativa da integralidade em saúde^10^, apesar do número reduzido de descrições sobre preocupação explícita com a proteção dos direitos das crianças, respostas das regiões Centro-Oeste, Sul e Sudeste trouxeram contribuições. Na primeira, o direito se materializou no acesso “à informação de forma facilitada tanto para as crianças quanto para os cuidadores, a partir de meios de comunicação diversos (rádio, redes sociais, outros aplicativos de comunicação), contribuindo para conscientização e promoção de saúde e bem-estar; promoção do lazer e de atividades físicas” (município da região Centro-Oeste). No município da região Sul, um dos equipamentos que auxiliam nessa garantia é o Centro de Referência de Assistência Social, por atuar nas vulnerabilidades sociais das famílias, além da conscientização dos profissionais de saúde quanto ao aumento da prevalência da obesidade infantil e à necessidade de intervir. Na região Sudeste, um dos relatos indicou a dificuldade de acesso aos equipamentos de saúde e a continuidade do cuidado como uma limitação.

### Eixo das Finalidades

Apenas um município da região Sudeste e outro da região Sul descreveram a integração de atividades de promoção e proteção da saúde, tratamento e recuperação. O município da região Sudeste apresenta serviço ambulatorial de obesidade, que estabelece comunicação com as unidades básicas de saúde, por meio do encaminhamento da criança e a contrarreferência para a APS. Todo o cuidado relativo à obesidade infantil é canalizado para um ambulatório de obesidade.

O município da região Sul apresentou uma perspectiva diferenciada de organização e integração do cuidado, uma vez que implementou a LCSO, na qual diversos equipamentos de saúde/sociais estão articulados. O cuidado nos demais municípios apresenta caráter assistencial, com priorização de atendimentos clínicos, desenvolvidos majoritariamente por nutricionistas. As ações de promoção da saúde da criança e prevenção da obesidade infantil ocorrem de maneira avulsa e concentradas no Programa Crescer Saudável. Tal desarticulação das ações, tornando-as “soltas e sem alinhamento” (município da região Sudeste), “descontínuas e de execução pontual e isolada” (município da região Sul), foi justificada pela insuficiência de profissionais.

A fragmentação do cuidado, com a atenção à saúde desempenhada mediante uma somatória de ações especializadas e desassociadas, foi elencada por Moura e Recine^24^ como um dos obstáculos para a prática da integralidade no SUS. Para que as ações em saúde operem de maneira integrada, coordenadas por um fluxo de atenção, devem estar fundamentadas pela longitudinalidade do cuidado, atingida pela continuidade, de modo permanente e consistente do cuidado, acompanhando as transformações das práticas em saúde^21^.

Segundo Rajjo et al.^20^, resultados significativos no cuidado em obesidade infantil são consequência de programas integrados e longitudinais de manejo no modo de vida das crianças. Três municípios participantes (regiões Centro-Oeste, Nordeste e Sul) desse estudo disseram que o exercício prático da atenção longitudinal está diretamente dependente do vínculo criado e do interesse da criança e da família em dar continuidade ao cuidado.

Outros fatores que dificultaram a concretização da longitudinalidade foram a alta demanda e número reduzido de profissionais e a não compreensão da lógica da atenção primária pela ESF^25^, enquanto coordenadora do cuidado.

### Eixo das Articulações

Adota-se, neste estudo, a intersetorialidade e a articulação multiprofissional como componentes do trabalho coletivo, essenciais para o exercício do cuidado integral, assim como a interdisciplinaridade – ainda que não evidenciada nas respostas dos municípios.

A interdisciplinaridade procura encontrar os elos que ligam uma área e seus fundamentos historicizados a outra, buscando ampliar compreensões e práticas^26^. Por multiprofissional, compreende-se a presença de diferentes profissionais trabalhando em ações compartilhadas^25^. Assim, a articulação multiprofissional pode ou não culminar em transformações nas práticas profissionais, diferente da vertente interdisciplinar. Por fim, a intersetorialidade é explicitada em ações nas quais diferentes setores sociais colaboram para o alcance de uma meta comum, mediante uma estreita coordenação de suas contribuições^27^.

O entendimento dos participantes sobre a interdisciplinaridade confundiu-se com o caráter multiprofissional. Em municípios das regiões Centro-Oeste e Nordeste, apesar de citarem que há planejamento interdisciplinar e multiprofissional, suas justificativas abrangeram apenas a perspectiva multiprofissional.

Um dos municípios da região Nordeste salientou que planejar “as ações com o auxílio intersetorial e multiprofissional têm mais chances de chegar ao resultado esperado, pois cada profissional ali envolvido irá somar no combate à obesidade”. Os trabalhos coletivos decorreram de discussões de casos em equipe, grupos terapêuticos e reuniões de matriciamento.

Em contrapartida, a região Norte explicitou que nem sempre o trabalho multiprofissional será suficiente, já que o planejamento pode ocorrer de maneira desarticulada. A ausência de equipes multiprofissionais qualificadas e regularmente capacitadas é uma das barreiras mais frequentes para efetivação da atenção integral a crianças com excesso de peso^20,28^.

A participação de profissionais da educação, da saúde, crianças e seus responsáveis na definição das ações direcionadas à obesidade infantil foi outro tópico estudado. Observou-se que a coordenação e o planejamento são realizados pelos profissionais de saúde. Em um município da região Sudeste, somente o nutricionista participa do planejamento. A presença dos profissionais da educação foi elencada nas regiões Centro-Oeste, Nordeste e Sul durante o planejamento, a execução e a aprovação pelos gestores da Secretaria de Educação.

A articulação intersetorial ocorreu, principalmente, com o setor da educação, por meio das avaliações antropométricas e ações educativas. Como exceção, um dos municípios da região Sul enalteceu a importância do Conselho Municipal de Segurança Alimentar e Nutricional enquanto facilitador do diálogo entre setores – saúde, educação, assistência social, esporte e segurança alimentar e nutricional – e sua constituição que engloba a sociedade civil.

Em nove municípios, houve dificuldade de articulação e priorização da obesidade infantil na agenda da saúde. A não priorização decorreu: da falta de implementação de protocolos e fluxos na rede de atenção; da insuficiência de recursos humanos, priorização de atendimentos individualizados de casos mais críticos, concentração de ações de prevenção no PSE; e do desinteresse dos profissionais de saúde. Tal cenário expressa engajamento insuficiente pelas partes envolvidas no cuidado em obesidade infantil. Assim, enfatiza que a integralidade enfrenta barreiras em termos de governança, tendo em vista a incipiente comunicação e a cooperação entre os diferentes setores e esferas de gestão^22^. Onde houve priorização do atendimento e do cuidado para crianças com obesidade, o fortalecimento veio com a implementação da LCSO.

O engajamento das partes envolvidas também está atrelado à corresponsabilização no cuidado. Apesar de o planejamento das ações restringir-se a alguns grupos, a corresponsabilidade se dá com atuação dos gestores e profissionais de saúde e educação. Os municípios das regiões Centro-Oeste, Nordeste, Norte, um da região Sudeste e um da Sul citaram a participação ativa das crianças e dos familiares no processo de cuidado.

Tal perspectiva corrobora práticas integrais, uma vez que há reconhecimento da necessidade de criação de vínculo, responsabilização dos indivíduos que participam desse longo processo, considerando as formas como as crianças e seus familiares expressam as suas necessidades de saúde^10^.

### Eixo das Interações

Diferentes abordagens utilizadas pelos profissionais de saúde, no âmbito individual e coletivo, podem exercitar aspectos do cuidado integral que incluem a necessidade de: ouvir mais, para compreender as singularidades, subjetividades e o contexto no qual os indivíduos e coletividades estão imersos; ver mais, estar atento aos gestos, ao olhar; e tocar mais, não só o local onde dói, mas no local onde há desconforto, preocupação, como potência para o cuidado^9^.

Em todos os municípios estudados, o exercício da escuta qualificada e da clínica ampliada ocorreu tanto em nível individual quanto coletivo. Um município da região Nordeste e um da região Sul relataram que tais estratégias “auxiliam a desvendar as necessidades das crianças de forma mais ampla” (município da região Nordeste), “para definir da melhor maneira o seu tratamento” (município da região Sul).

Tais estratégias podem fortalecer práticas integrais, uma vez que favorecem a participação ativa. Os trechos 1 e 2 descrevem modos como as abordagens foram aplicadas nos serviços de saúde, tanto na prática assistencial quanto em capacitações profissionais:

Trecho 1

Escuta qualificada e relações dialógicas estão presentes tanto no atendimento em grupo, como nas consultas individuais, sempre dando atenção à individualidade de cada paciente e suas demandas. A clínica ampliada ocorre com atendimento médico, e também com consultas pontuais de nutrição e psicologia, que servem para compreender a realidade de cada família e orientar de acordo com as demandas identificadas e levantadas pelas crianças e famílias (município da região Sul).

Trecho 2

(...) os conceitos citados [escuta qualificada e clínica ampliada] (...) são amplamente discutidos nas reuniões de profissionais, especialmente com os nutricionistas que são os coordenadores do cuidado na LCSO. Esses profissionais são responsáveis por capacitar as equipes em relação ao cuidado do usuário com obesidade (município da região Nordeste).

É imprescindível o papel ativo dos diferentes sujeitos em ações de cuidado à obesidade infantil, via estabelecimento de relações dialógicas. Segundo Freire^29^, essas interações configuram momentos para refletir sobre problemáticas vivenciadas, no sentido de superá-las, considerando os diferentes saberes e as perspectivas daqueles que participam do cuidado em saúde.

Todos os participantes disseram que há intercâmbio entre conhecimentos técnicos e práticos, mas com características diferenciadas. O município da região Norte citou que essa prática ocorre eventualmente e depende da iniciativa do profissional. Um município da região Sudeste ressaltou a necessidade de apoio da gestão para que haja maior sensibilização quanto à importância dessas trocas.

Apenas a região Sul trouxe detalhamentos sobre tais interlocuções. Majoritariamente, decorrem de atividades grupais com condução de diferentes profissionais da saúde, estabelecendo parcerias intersetoriais com a participação ou não de crianças e de seus responsáveis. As trocas nesses espaços ocorrem de modo dialógico, sendo potentes na compreensão de fatores que impactam no desenvolvimento da obesidade em um grupo específico, contribuindo para ampliação da resolutividade. Destacou-se o protagonismo das crianças, apesar de costumeiramente serem excluídas e silenciadas. Segundo Ehrich *et al.*^30^, incluir a criança ativamente pode aprimorar o seu conhecimento, a sua autoconfiança, sua imaginação e sua confiabilidade no profissional de saúde, contribuindo para a sua autonomia e sua efetividade do cuidado integral.

No que tange às limitações do estudo, ressalta-se que foram recebidos questionários com preenchimento incompleto e houve dificuldade de contato nas regiões Centro-Oeste e Norte. Entendemos que o período pandêmico foi um fator que limitou o detalhamento das ações e a execução de atividades desenvolvidas no contexto da obesidade infantil.

## CONCLUSÕES

A integralidade configura-se como um horizonte a ser alcançado na prática do cuidado da obesidade infantil, é almejada enquanto orientadora da organização dos serviços dos municípios estudados, mas ainda enfrenta desafios para sua efetivação.

Enquanto potências para o cuidado integral, observou-se: a oferta de serviços em diferentes níveis de atenção; a relevância do PSE e do Programa Crescer Saudável no desenvolvimento de ações voltadas à multidimensionalidade da obesidade infantil; as estratégias de sistematização do cuidado, como a implementação da LCSO e a ampliação do diálogo e da humanização nas práticas em saúde; e a articulação intersetorial para criação de respostas adequadas às necessidades ampliadas das crianças e suas famílias.

Por fim, para que o cuidado da obesidade infantil seja capaz de provocar transformações na realidade de diferentes sujeitos, é preciso entendê-lo na sua complexidade, e isso está diretamente relacionado ao olhar para a integralidade em saúde e sua dinamicidade. Isso inclui (re)pensar políticas públicas, práticas profissionais, organizações dos processos de trabalho para que sejam, de fato, mais inclusivas, participativas, dialógicas, humanizadas, solidárias, justas e, portanto, eficazes. Nesse sentido, há a necessidade de se mobilizar diferentes setores/espaços/sujeitos em prol dessa agenda.
